# Propionate metabolism in *Desulfurella acetivorans*

**DOI:** 10.3389/fmicb.2025.1545849

**Published:** 2025-02-12

**Authors:** Eugenio Pettinato, Thomas M. Steiner, Eric A. Cassens, Thomas Geisberger, Christian Seitz, Simone König, Wolfgang Eisenreich, Ivan A. Berg

**Affiliations:** ^1^Institute for Molecular Microbiology and Biotechnology, University of Münster, Münster, Germany; ^2^Bavarian NMR Center–Structural Membrane Biochemistry, Department of Chemistry, Technische Universität München, Garching, Germany; ^3^Core Unit Proteomics, Interdisciplinary Center for Clinical Research, Faculty of Medical, University of Münster, Münster, Germany

**Keywords:** propionate assimilation, tricarboxylic acid cycle, *Desulfurella acetivorans*, methylcitrate cycle, methylmalonyl-CoA pathway

## Abstract

*Desulfurella acetivorans* is a strictly anaerobic sulfur-reducing deltaproteobacterium that can grow heterotrophically by oxidation of acetate or autotrophically with molecular hydrogen. Here we show that *D. acetivorans* possesses a putative operon encoding enzymes of the methylcitrate cycle of propionate oxidation and demonstrate that this bacterium is capable of propionate growth. However, activities of the methylcitrate cycle enzymes could not be detected in extracts of propionate-grown cells, and experiments with [U-^13^C_3_]propionate and comparative proteomic analysis of acetate- and propionate-grown cells suggested that the methylcitrate cycle is not active during propionate growth. Instead, propionyl-CoA assimilation proceeds via its carboxylation to methylmalonyl-CoA, which is further converted to succinyl-CoA. The latter is directed to the tricarboxylic acid (TCA) cycle, where it is converted to oxaloacetate and condenses with acetyl-CoA (produced by decarboxylation of another oxaloacetate molecule) to form citrate, which is oxidized in the TCA cycle. These results highlight the uncertainty of genomic predictions in the analysis of microbial metabolic pathways and the need for their experimental confirmation.

## Introduction

**Figure 1:**
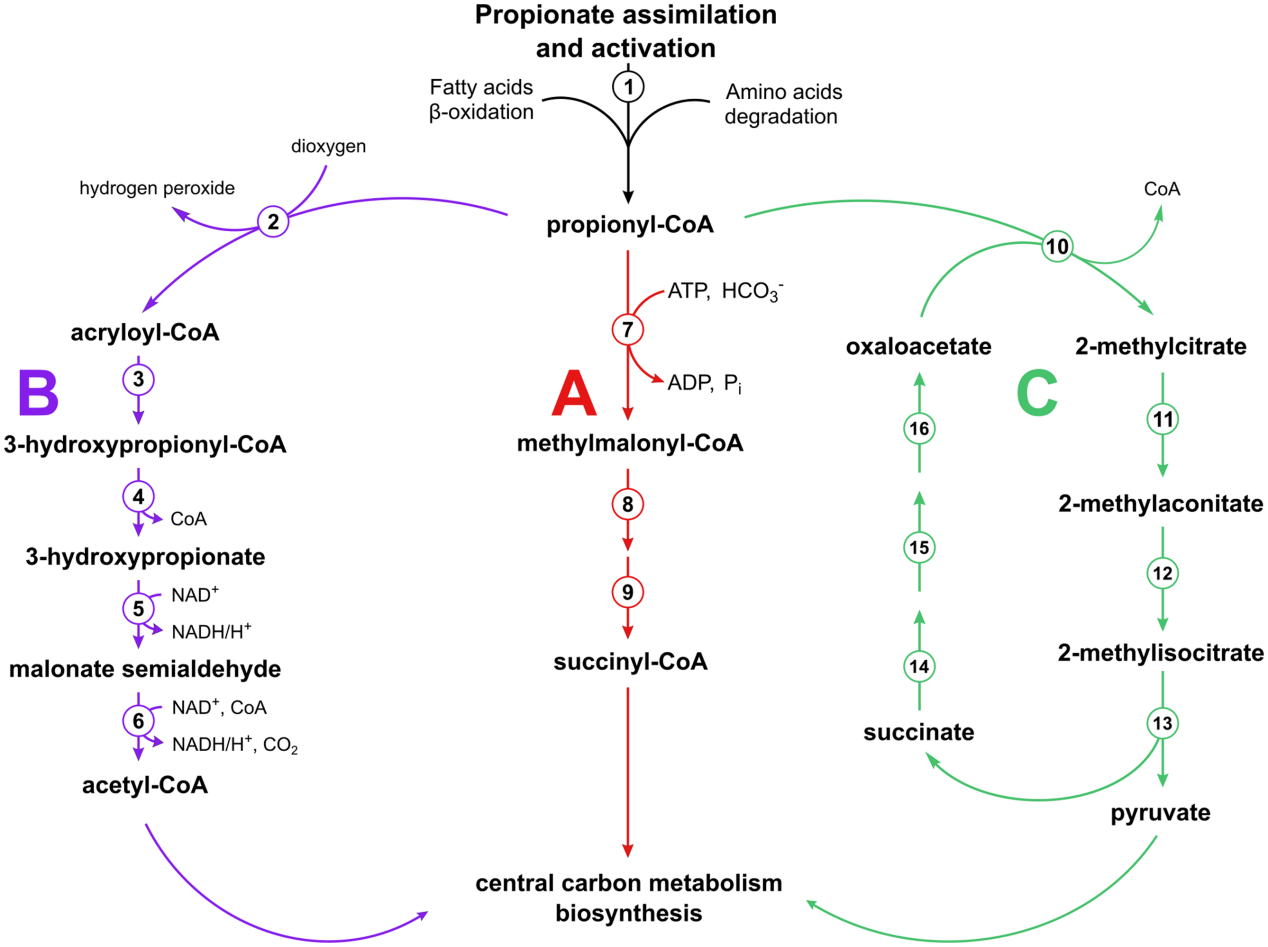
Pathways of propionyl-CoA utilization functioning in various organisms. The methylmalonyl-CoA pathway (A) with enzymes numbered (1) AMP-dependent synthetase, (7) propionyl-CoA carboxylase, (8) methylmalonyl-CoA epimerase, (9) methylmalonyl-CoA mutase; the modified β-oxidation pathway (B) (Otzen et al., 2014) with the enzymes involved numbered (2) propionyl-CoA oxidase, (3) acryloyl-CoA hydratase, (4) 3-hydroxypropionyl-CoA hydrolase, (5) 3-hydroxypropionate dehydrogenase, (6) malonate semialdehyde dehydrogenase; and the methylcitrate cycle (C) (Horswill and Escalante-Semerena, 1999, 2001) with the enzymes involved numbered (10) 2-methycitrate synthase, (11) 2-methylcitrate dehydratase, (12) aconitase, (13) 2-methylisocitrate lyase, (14) succinate dehydrogenase, (15) fumarase, (16) malate dehydrogenase.

Propionate is a short-chain fatty acid commonly found in nature as a fermentation end product of the metabolism of a variety of microorganisms. It can be encountered in the industrial field where it is used in the production of biopolymers (Policastro et al., 2021) or as a food preservative, due to its antimicrobial activity (Salmond et al., 1984; Brock and Buckel, 2004). Propionate (in its activated form, propionyl-CoA) is an intermediate in some central metabolic pathways (Erb et al., 2007; Zarzycki et al., 2009; Berg, 2011; Khomyakova et al., 2011; Fuchs and Berg, 2014; Borjian et al., 2016) and is also produced during the degradation of some amino acids and the beta-oxidation of odd-chain fatty acids (Nelson and Cox, 2017). Consequently, different pathways have evolved that allow propionyl-CoA utilization (Figure 1). Animals and many microorganisms use the methylmalonyl-CoA pathway starting with propionyl-CoA carboxylation (or transcarboxylation) to (*S*)-methylmalonyl-CoA. The latter compound is converted to (*R*)-methylmalonyl-CoA and then to succinyl-CoA, a tricarboxylic acid (TCA) cycle intermediate (Knight, 1962; Olsen and Merrick, 1968; Halarnkar and Blomquist, 1989; Nelson and Cox, 2017; Westerholm et al., 2022). Plants and some microorganisms metabolize propionyl-CoA to acetyl-CoA via 3-hydroxypropionate (modified ß-oxidation pathway) (Lucas et al., 2007; Otzen et al., 2014), whereas most of fungi and bacteria use the methylcitrate cycle (Tabuchi et al., 1974; Textor et al., 1997; Gerike et al., 1998; Horswill and Escalante-Semerena, 1999), which is a modified glyoxylate cycle that starts with the condensation of oxaloacetate with propionyl-CoA (instead of acetyl-CoA) catalyzed by methylcitrate synthase. Methylcitrate is then converted to methylisocitrate and finally cleaved into pyruvate, the product of the cycle, and succinate. The latter compound is converted back into oxaloacetate, closing the cycle (Figure 1). To date, this cycle has only been shown in bacteria grown under aerobic conditions, likely due to the potential accumulation of toxic 2-methycitrate in cells if succinate oxidation in the cycle is impaired in the absence of oxygen (Simonte et al., 2017). Nevertheless, the possibility of the functioning of this cycle under anaerobic conditions has been proven, as *E. coli* could grow on propionate via nitrate respiration after deletion of a post-translational regulatory system affecting the expression of the methylcitrate cycle operon (Simonte et al., 2017). Other anaerobic propionate utilization pathways have been proposed based on the results of ^13^C labeling analyses, but no supporting biochemical data have yet been presented (Tholozan et al., 1988; de Bok et al., 2001).

*Desulfurella acetivorans* is a strictly anaerobic thermophilic sulfur-reducing bacterium of the phylum Campylobacterota capable of heterotrophic growth through acetate oxidation by means of the oxidative TCA (oTCA) cycle, or of autotrophic CO_2_ fixation through the reversed oxidative TCA (roTCA) cycle (Bonch-Osmolovskaya et al., 1990; Schmitz et al., 1990; Pradella et al., 1998; Mall et al., 2018; Steffens et al., 2021, 2022), which is a variant of the reductive TCA (rTCA) cycle. One of the most challenging steps in the roTCA cycle is represented by citrate cleavage catalyzed by *Si*-citrate synthase (Nunoura et al., 2018; Mall et al., 2018), whereas ATP-dependent citrate lyase is typically responsible for this reaction in the standard variant of the rTCA cycle (Ivanovsky et al., 1980; Buchanan et al., 2017). *D. acetivorans* possesses three different *Si*-citrate synthase isoforms (i.e., AHF97591, AHF97477, AHF97305), which raised the question of whether these isoenzymes have evolved to operate in a specific direction of the cycle. To answer this question, we heterologously produced and characterized the three isoenzymes. The *AHF97477* gene product was found to be the main *Si*-citrate synthase in *D. acetivorans* under both autotrophic and heterotrophic conditions (Steffens et al., 2021). The *AHF97305* gene product was purified and obtained in a soluble form, but its activity as a citrate synthase was never demonstrated, and the function of this enzyme remains unknown. Here, we have heterologously produced the third citrate synthase homolog (AHF97591) in an active form and characterized it. To our surprise, this enzyme could utilize not only acetyl-CoA but also propionyl-CoA as substrate, forming 2-methylcitrate as product, thus being a promiscuous citrate/2-methylcitrate synthase, a key enzyme of the 2-methylcitrate cycle of propionate oxidation. The presence of this enzyme in the *D. acetivorans* genome suggests the functioning of the methylcitrate cycle in sulfur oxidizers, even though *D. acetivorans* has been described as not being capable of using propionate as a carbon source (Bonch-Osmolovskaya et al., 1990). Here we show that *D. acetivorans* is able to grow on propionate and possesses in its genome a methylcitrate cycle operon that encodes functional enzymes. Nevertheless, it still uses the methylmalonyl-CoA pathway for propionate utilization. This highlights the uncertainty of genomic predictions in the analysis of microbial metabolic pathways.

## Materials and methods

### Chemicals

DL-threo-2-methylisocitrate was obtained from Adooq Bioscience LLC. (Irvine, CA, USA). Other chemicals, biochemicals and Neubauer counting chambers were obtained from Sigma-Aldrich, Merck, Roth, VWR or AppliChem. ^13^C-labeled chemicals were obtained from Eurisotop. Materials for molecular biology were purchased from New England BioLabs. Materials and equipment for protein purification were obtained from GE Healthcare, Macherey-Nagel or Millipore. Lead acetate paper was obtained from Macherey-Nagel. Primers for were synthesized by Sigma-Aldrich.

### Microbial strains and growth conditions

*Desulfurella acetivorans* A63 (DSM 5264) was obtained from the Deutsche Sammlung von Mikroorganismen und Zellkulturen (DSMZ). The cells were grown autotrophically (with H_2_/CO_2_) or heterotrophically in medium containing 6.2 mM NH_4_Cl, 2.2 mM CaCl_2_·2H_2_O, 1.6 mM MgCl_2_·6H_2_O, 4.4 mM KCl, 1.7 mM KH_2_PO_4_, 1.3 mM K_2_HPO_4_, 10 g l^−1^ sulfur powder, 1 mL l^−1^ SL-10 trace element solution, and 1 mL l^−1^ Wolfe’s vitamin solution. The trace element solution contained 3 g l^−1^ FeCl_2_·4H_2_O, 70 mg l^−1^ ZnCl_2_, 100 mg l^−1^ MnCl_2_·4H_2_O, 4 mg l^−1^ CuCl_2_·2H_2_O, 24 mg l^−1^ NiCl_2_·6H_2_O, 36 mg l^−1^ Na_2_MoO_4_·2H_2_O, 30 mg l^−1^ H_3_BO_3_, 224 mg l^−1^ CoCl_2_·6H_2_O. The vitamin solution contained 20 mg l^−1^ biotin, 20 mg l^−1^ folic acid, 100 mg l^−1^ pyridoxamine dihydrochloride, 50 mg l^−1^ thiamine dihydrochloride, 50 mg l^−1^ riboflavin, 50 mg l^−1^ nicotinic acid, 50 mg l^−1^ DL-Ca-pantothenate, 1 mg l^−1^ cyanocobalamin, 50 mg l^−1^ 4-aminobenzoic acid and 50 mg l^−1^ lipoic acid. The medium was prepared without sulfur and vitamin solutions, made anaerobic by applying gassing/degassing cycles with N_2_ (100%) for approximately 45–60 min per liter of medium (4 min for one cycle), and dispensed anaerobically into serum bottles containing sulfur powder. The bottles were sealed with butyl rubber stoppers and aluminum caps and autoclaved for 40 min at 110°C. Before inoculation, the medium was reduced by the addition of Na_2_S·9H_2_O to a final concentration of 0.05% (w/v) and supplemented with vitamins. When cultivated heterotrophically, sodium acetate or sodium propionate was supplemented as sole carbon source to a final concentration of 0.5 and 0.05%, respectively, corresponding to 52 mM and 5.2 mM propionate. The gas phase was replaced with N_2_/CO_2_ (80:20, v/v) (heterotrophic growth) or H_2_/CO_2_ (80:20, v/v) (autotrophic growth) at 1 bar overpressure. pH was adjusted with sodium bicarbonate to 6.8–7.0. Cultures were incubated at 55°C while shaking at 130 rpm.

*Escherichia coli* strains Top10, Rosetta 2 (DE3), C41 (DE3) were grown at 37°C in lysogeny broth (LB) medium. If necessary, antibiotics were added to the cultures to a final concentration of 100 μg ml^−1^ ampicillin, 50 μg ml^−1^ kanamycin, and 34 μg ml^−1^ chloramphenicol.

### Cell extract preparation

*D. acetivorans* cultures (200 mL, typically 10^7^ to 10^8^ cells/ml) were filtered aerobically (Whatman GF/D, 47 mm) to remove the elemental sulfur before centrifugation (15,000 × *g*, 4°C, 20 min). The supernatant was discarded gently, and the resulting cell pellet was resuspended with 20 mL of fresh sulfur-free medium. The cell suspension was transferred to a 50 mL centrifuge tube and centrifuged again (21,000 × *g*, 4°C, 20 min). After having discarded the supernatant, the cells were frozen with liquid nitrogen and stored at −80°C for up to 6 months.

To perform enzymatic assays, the frozen cells were thawed on ice and resuspended in 20 mM Tris–HCl (pH 7.8 at 55°C), 5 mM dithiothreitol (DTT), and lysed on ice with an ultrasonic homogenizer (60% amplitude, 4 min, 1-s pulse, 2-s breaks; total energy input 2,000 kJ). The insoluble cell debris was removed by centrifugation (21,000 × *g*, 4°C, 20 min).

### CoA-thioesters synthesis

Acetyl-CoA, propionyl-CoA and succinyl-CoA were synthesized from the corresponding anhydrides and CoA according to Simon and Shemin (1953).

### Cloning of *D. acetivorans* AHF97591 and AHF97589 in *E. coli*

Standard protocols were used for the purification, cloning, transformation and amplification of DNA.

The *AHF97591* (*Si*-citrate/2-methylcitrate synthase) encoding gene was amplified by PCR with Q5 High-Fidelity DNA Polymerase using a forward primer (5′-GCGTAGAATTCCATG AAGCTTAAAGAAAAAC-3′) introducing an EcoRI site (bold) and a reverse primer (5′-ATTATAGTCGACTTAAAACCACACTTTAGCT-3′) introducing a SalI site (bold). PCR conditions were as follows: 35 cycles of 10 s denaturation at 98°C, 30 s primer annealing at 66°C, and 180 s elongation at 72°C. The isolated PCR product was treated with the corresponding restrictases and ligated into the expression vector pET16b containing a sequence encoding a N-terminal His_6_-tag.

The *AHF97589* (methylcitrate dehydratase) encoding gene was amplified by PCR with Q5 High-Fidelity DNA Polymerase using a forward primer (5′-GGGACATATGGATAAGTACACTAAAACTTTTGCA-3′) introducing a NdeI site (bold) and a reverse primer (5′-GGAAGGATCCTTATACCCTCAATAATTGAGCTAATTTAG-3′) introducing a BamHI site (bold). PCR conditions were as follows: 30 cycles of 30 s denaturation at 98°C, 30 s primer annealing at 60°C, and 45 s elongation at 72°C. The isolated PCR product was treated with the corresponding restrictases and ligated into the expression vector pET16b containing a sequence encoding a N-terminal His_10_-tag. The plasmids were transformed into *E. coli* TOP10 for amplification, followed by purification and sequencing.

The *AHF97590* (methylisocitrate lyase) encoding gene was amplified by PCR with Q5 High-Fidelity DNA Polymerase using a forward primer (5′-AAAACATATGAGCTGGCTTGTAGAAGAACGTATG-3′) introducing an NdeI site (bold) and a reverse primer (5′-AAAAGGATCCTCATTTTTTTTCAAAGATAGCTTTGTC-3′) introducing a BamHI site (bold). PCR conditions were as follows: 35 cycles of 10 s denaturation at 98°C, 30 s primer annealing at 68°C, and 45 s elongation at 72°C. The isolated PCR product was treated with the corresponding restrictases and ligated into the expression vector pET16b containing a sequence encoding a N-terminal His_6_-tag.

### Heterologous expression in *E. coli*

The amplified expression vectors pET28b-AHF97591, pET16b-AHF97589 and pET16b-AHF97590 were used to transform *E. coli* Rosetta 2 (DE3), *E. coli* C41 (DE3), and *E. coli* BL21, respectively. The cells were grown at 37°C in LB medium containing 10 g l^−1^ NaCl, 5 g l^−1^ yeast extract, and 10 g l^−1^ bacto-tryptone with kanamycin and chloramphenicol (pET28b) or ampicillin and chloramphenicol (pET16b). Expression was induced at an optical density (OD_600_ nm) of 0.5–0.8 with 1 mM isopropyl-ß-D-thiogalactopyranoside (IPTG), and the temperature was lowered to 20°C. The cells were harvested after overnight growth and stored at −20°C until use.

### Preparation of *E. coli* cell extracts

Frozen cells expressing *AHF97591* and *AHF97589* genes were suspended in a triple volume of 20 mM Tris–HCl (pH 7.8), containing 500 mM NaCl, and 20 mM imidazole, and 0.1 mg ml^−1^ DNase I. The cell suspensions were lysed by a threefold passage through a chilled French pressure cell (103 MPa). In the case of AHF97589 the supernatant (cell extract) has been subjected to a heat precipitation step (55°C; 15 min) to remove thermally unstable *E. coli* proteins. The resulting cell lysate was centrifuged (100,000 x *g*; 4°C; 60 min), filtered and used for protein purification.

Frozen cells expressing *AHF97590* gene were suspended in a double volume of 20 mM Tris–HCl (pH 7.8), containing 300 mM NaCl, and 10 mM imidazole. The cells were disrupted using the SONOPULS HD 2070.2 ultrasonic homogenizer (BANDELIN, Berlin, Germany) with the standard horn SH 70 G (BANDELIN). Cell disruption was performed 5 times for 4 min (1 s ultrasonic pulse followed by a 2 s pause) with an amplitude of 80% and a 5-min-pause separating each run. The resulting cell lysate was centrifuged (20,000 x *g*; 4°C; 15 min), filtered through a 0.4 μm syringe filter and used for protein purification.

### Purification of recombinant proteins

The heterologously produced His-tagged methylcitrate synthase (AHF97591) and methylcitrate dehydratase (AHF97589) enzymes were purified using affinity chromatography. The corresponding cell extracts were applied to a Protino Ni-NTA column (Macherey-Nagel) that had been equilibrated with 20 mM Tris–HCl (pH 7.8) containing 500 mM NaCl and 20 mM imidazole. In the case of methylcitrate synthase (AHF97591), the column was washed with the same buffer containing 35 mM imidazole, then 70 mM imidazole. In the case of methylcitrate dehydratase (AHF97589), the column was washed with the same buffer containing 50 mM imidazole. The recombinant enzymes were eluted with the same buffer containing 300 mM imidazole. The enzyme was concentrated using a 10 K Vivaspin Turbo 4 and stored at −20°C with glycerol (50%, v/v). The identity of the purified recombinant protein was confirmed at the IZKF Core Unit Proteomics Münster with tryptic in-gel digestion and mass spectrometric analysis using Synapt G2 Si coupled to M-Class (Waters Corp.).

For the purification of the heterologously produced His-tagged methylisocitrate lyase (AHF97590), the corresponding cell extract was applied to a Protino Ni-NTA column (Macherey-Nagel) that had been equilibrated with 20 mM Tris–HCl (pH 7.5) containing 300 mM NaCl and 10 mM imidazole. The column was washed with the same buffer with imidazole concentration increased in 25 mM steps. The protein eluted at 300 mM imidazole. The collected enzyme fraction was concentrated using a 10 K Vivaspin Turbo 4 and further purified by size exclusion chromatography using the Superdex® 200 Increase 10/300 GL column (SEC, Merk KGaG, Darmstadt, Germany), concentrated using a 10 K Vivaspin Turbo 4 and stored at −80°C with glycerol (20%, v/v) until use.

### Enzyme assays

Continuous enzyme assays were performed spectrophotometrically (0.3 mL reaction mixture) with glass (for visible light) cuvette, discontinuous enzyme assays were performed with HPLC with samples taken at different time points. All enzyme activities were tested at 55°C. Reactions following CoA formation with DTNB were measured at 412 nm (ε_412_ = 14.2 mM^−1^ cm^−1^; Riddles et al., 1979). Reactions involving NAD(P)H were measured at 365 nm (ε_NADH_ = 3.4 mM^−1^ cm^−1^, ε_NADPH_ = 3.5 mM^−1^ cm^−1^; Bergmeyer, 1975). Reactions involving phenylhydrazine were measure at 324 nm (ε_glyoxylate-phenylhydrazone_ = 17 mM^−1^ cm^−1^, Herter et al., 2001).

Citrate synthase was measured spectrophotometrically at 412 nm as the oxaloacetate-dependent formation of free CoA from acetyl-CoA. The reaction mixture contained 100 mM Tris–HCl (pH 7.8), 5 mM oxaloacetate, 1 mM acetyl-CoA, 1 mM DTNB, and cell extract.

Methylcitrate synthase was measured in the same way as citrate synthase, but acetyl-CoA was replaced by propionyl-CoA.

Malate dehydrogenase was measured spectrophotometrically at 365 nm as the oxaloacetate-dependent oxidation of NADH. The assay mixtures contained 100 mM Tris–HCl (pH 7.8), 5 mM DTE, 5 mM MgCl_2_, 0.5 mM NADH, 5 mM oxaloacetate, and cell extract.

Isocitrate dehydrogenase was measured spectrophotometrically at 365 nm as the isocitrate-dependent reduction of NADP. The assay mixture contained 100 mM Tris–HCl (pH 7.8), 5 mM DTE, 5 mM MgCl_2_, 1 mM NADP, 10 mM DL-isocitrate, and cell extract.

Isocitrate lyase was measured as the isocitrate-dependent formation of phenylhydrazone glyoxylate. The reaction mixture contained 100 mM Tris–HCl (pH 7.8), 5 mM DTE, 5 mM MgCl_2_, 3.5 mM phenylhydrazine, 10 mM DL-isocitrate, and cell extract.

Methylsocitrate lyase activity was measured as the methylisocitrate-dependent formation of phenylhydrazone glyoxylate. The reaction mixture contained 100 mM MOPS-KOH (pH 7.5), 5 mM DTT, 5 mM MgCl_2_, and 3.5 mM phenylhydrazine, 0.5 mM *threo*-methylisocitrate, and purified enzyme. After the addition of the protein, the reaction mixture was incubated at 55°C for up to 10 min while shaking at 400 rpm. The assay was stopped by adding the same volume of 1 M HCl, and the formation of pyruvate phenylhydrazone was measured by UHPLC following absorbance at 324 nm. The concentration of pyruvate was determined using a calibration curve generated in a similar manner with pyruvate. The activity of the backward reaction (i.e., methylisocitrate formation) was measured in reaction mixture containing 100 mM MOPS-KOH (pH 7.5), 100 mM pyruvate, 100 mM succinate, 5 mM DTT, and 5 mM MgCl_2_. The reaction was started by addition of the purified protein and incubated for 24 h at 55°C while shaking at 400 rpm. The assay was stopped by the addition of the same volume of 1 M HCl, and the product formation was measured by HPLC-MS at 205 m/z in negative mode.

Methylcitrate dehydratase activity of the AHF97589-gene-product was proved via GC–MS following the formation of methylaconitate. The reaction mixture contained 100 mM Tris–HCl, 1 mM 2-methylcitrate, and purified heterologously produced protein (AHF97589-gene-product).

All the CoA-transferase activities were measured via UHPLC and the reaction mixtures (40 μL) contained 100 mM Tris–HCl (pH 7.8), 5 mM MgCl_2_, 5 mM DTT, and cell extract. Activities were tested with acetyl-CoA, succinyl-CoA or propionyl-CoA toward acetate, succinate or propionate. The reaction was stopped after 1 min by the addition of 20 μL of 1 M HCl/10% acetonitrile (sample:stop solution, 1:1 [v/v]). The specific activities were calculated by considering the peaks of consumed and formed CoA-thioesters. The concentration of the latter was calculated by multiplying the starting substrate concentration by the relative abundance (%) of the formed CoA-thioester integrated peak area.

Succinyl-CoA synthetase activity was measured using UHPLC as the CoA-dependent formation of succinyl-CoA from succinate and CoA. The assay mixture contained 100 mM Tris–HCl (pH 7.8), 5 mM DTT, 5 mM MgCl_2_, 20 mM succinate, 5 mM ATP, 1 mM CoA, and cell extract.

Acetate kinase activity was measured spectrophotometrically following acetate-dependent oxidation of NADH at 365 nm in an assay containing (in 0.3 mL) 100 mM Tris–HCl (pH 7.8), 5 mM DTT, 5 mM MgCl_2_, 20 mM acetate, 5 mM ATP, 0.5 mM NADH, 5 mM PEP, 5 U pyruvate kinase (rabbit muscle, Sigma P9136), 25 U lactate dehydrogenase (rabbit muscle, Sigma L2500), and cell extract. The activity of AMP-forming acetyl-CoA synthetase and propionyl-CoA synthetase were tested by including 5 U myokinase and 0.5 mM CoA in the same reaction mixture. For measurement of propionyl-CoA synthetase activity, propionate (20 mM) was added in the reaction mixture instead of acetate.

Phosphotransacetylase activity was measured spectrophotometrically in an assay coupled with endogenous citrate synthase and malate dehydrogenase following NAD reduction at 365 nm as the CoA- and acetyl-phosphate dependent acetyl-CoA formation in the reaction mixture containing 100 mM Tris–HCl (7.8), 5 mM DTT, 5 mM NAD, 10 mM malate, 10 mM acetyl-phosphate, 1 mM CoA, and cell extract.

Propionyl-CoA carboxylase activity was proved via GC–MS following the time-dependent formation of [1-^13^C]methylmalonate (0, 5 and 10 min). The reaction mixture contained 100 mM Tris–HCl (pH 7.8), 5 mM MgCl_2_, 5 mM DTE, 5 mM ATP, 1 mM propionyl-CoA, 40 mM NaH^13^CO_3_, and cell extract of propionate-grown *D. acetivorans* cells.

### Preparation of samples for MS amino acid and carbonic acid analysis

Isolation of protein bound amino acids was done as described previously (Eylert et al., 2008). About 2 mg of bacterial sample (lyophilized cell pellet) was suspended in 500 μL of 6 M hydrochloric acid and hydrolyzed overnight at 105°C. The reaction mixture was dried under a stream of nitrogen. The residue was suspended in 200 μL of 50% acetic acid. Amino acids were isolated using a small column of Dowex 50 W X8 (7 × 10 mm; 200–400 mesh, 34–74 μm, H^+^-form). The column was first washed with 2 mL H_2_O, then amino acids were eluted with 1 mL 4 M aqueous ammonia solution. The ammonia eluate was dried under a stream of nitrogen at 70°C. The dried residue was treated with 50 μL of *N*-(*tert*-butyldimethylsilyl)-*N*-methyltrifluoroacetamide (MTBSTFA) containing 1% *N*-*tert*-butyldimethylsilylchloride (TBDMS) and 50 μL of anhydrous ACN at 70°C for 30 min. The TBDMS derivatives of amino acids were then analyzed by GC–MS. Acid hydrolysis leads to the conversion of glutamine and asparagine to glutamate and aspartate, respectively. Therefore, results given for aspartate and glutamate correspond to asparagine or aspartate and glutamine or glutamate, respectively. To account for different derivatization and ionization efficiency of each amino acid, an equimolar amino acid mixture (2.5 μM in 0.1 M HCl) was used to determine the response factor for each amino acid. Therefore, 200 μL of the amino acid mixture was dried under a stream of nitrogen at 70°C. The dried residue was treated with 50 μL MTBSTFA containing 1% TBDMS and 50 μL anhydrous ACN at 70°C for 30 min. The same method was used to prepare the samples for the detection of acetate and methylmalonate.

For the analysis of AHF97589 gene product activity as methylcitrate dehydratase, the samples were dried under a stream of nitrogen at room temperature. The dried residues were then derivatized for GC–MS analysis using two different conditions. Replicate A and controls were derivatized with 100 μL of *N*-methyl-*N*-(trimethylsilyl)trifluoroacetamide (MSTFA) and incubated for 90 min at 40°C while shaking (110 rpm). Replicate B was first treated with 100 μL of methoxyamine-hydrochloride in pyridine (15 mg/mL) at 40°C for 45 min and then dried under a nitrogen stream at room temperature. After addition of 100 μL of MSTFA to the dried residue, the samples were incubated for 90 min at 40°C while shaking (110 rpm). The product of the analyzed reaction, 2-methyl-*trans*-aconitate was commercially available and was used as a reference substance. The standard was treated and derivatized according to the two methods described above.

### GC–MS analysis

GC–MS analysis was performed with a QP2010 Plus gas chromatograph–mass spectrometer (Shimadzu) equipped with a fused silica capillary column (Equity TM-5; 30 m × 0.25 mm, 0.25-μm film thickness; SUPELCO) and a quadrupole detector working with electron impact ionization at 70 eV. An aliquot (0.1–6 μL) of the TBDMS-derivatized samples was injected in 1:5 split mode at an interface temperature of 260°C and a helium inlet pressure of 70 kPa.

For the analysis of ^13^C excess (percentage of labeled molecules) and isotopologue composition of bacterial amino acids, selected ion monitoring was used with a sampling rate of 0.5 s and LABSOLUTION software (Shimadzu) was used for data collection and analysis. Isotopologue calculations were performed for m/z [M - 57]^+^ or m/z [M - 85]^+^. For analysis of the relative amino acid composition in *D. acetivorans* protein, measurements were performed in scan mode in a mass range from 45 m/z to 700 m/z with an injection volume of 0.1 μL.

For amino acids, the column was heated to 150°C and kept at 150°C for 3 min, after which was heated to 280°C (7°C per min) and held at that temperature for 3 min. The injector temperature was 260°C. Measurements were performed in SCAN mode with a scan interval of 0.5 s and a mass range of 50–600 m/z. All samples were measured three times for technical replicates. The calculation of ^13^C excess was done as described previously (Eylert et al., 2008) and comprises (1) the detection of GC–MS spectra of unlabeled derivatized metabolites; (2) the determination of the absolute mass of isotopologue enrichments and distributions of labeled metabolites of the experiment; and (3) the correction of the absolute ^13^C incorporation by subtracting the contributions of the heavy isotopologues due to the natural abundances in the derivatized metabolites to calculate the enrichments and distributions of the isotopologues. For the ^13^C-labeling data analysis, Isotopo-4 software was used (Ahmed et al., 2014).

For samples containing 2-methylaconitate (i.e., 2-methylcitrate dehydratase activity test), the column was held at 150°C for 3 min followed by a temperature gradient of 10°C min^−1^ to a final temperature of 280°C. Measurements were performed in SCAN-mode with a mass range of 50–700 m/z. Identification of products in the assay was done through database comparison using NIST14 database, as well as through comparison to the refence substance, 2-methylaconitate.

### LC–MS analysis of the product of methylisocitrate lyase reaction

UHPLC-UV/MS analysis was performed on the ACQUITY Premier system (Waters Cooperation, Milford, Massachusetts, USA) using the Atlantis™ PREMIER BEH C18 AX column. A gradient method recommended by the supplier was used to separate di- and tricarboxylic acids (Supplementary Table 2). The following buffers were used: A, 50 mM ammonium formate and 0.9% formic acid in water (pH 2.9), B, 0.9% formic acid in acetonitrile, and C, 0.9% formic acid in water. The flow rate was 0.35 mL/min, the column temperature was 30°C. The mass detector was run in negative ionization mode with a 0.8 kV capillary voltage, 5 V cone voltage, and 600°C probe temperature.

### Pyruvate phenylhydrazone determination

The concentration of pyruvate formed in the methylisocitrate lyase reaction was determined by UPLC following pyruvate phenylhydrazone formation at 324 nm. The analysis was performed on the Agilent 1290 Infinity II LC system (Agilent Technologies, Inc., Santa Clara, USA) using the NUCLEOSHELL Bluebird RP 18 column (MACHEREY-NAGEL GmbH & Co. KG, Düren, Germany). Separation was performed using an isocratic method at a flow rate of 0.4 mL/min for 6 min, with the column heated to 30°C. The KH_2_PO_4_/H_3_PO_4_ buffer contained 6.5 g/L KH_2_PO_4_, with pH being adjusted to pH 2.5 with H_3_PO_4_. Quantification of pyruvate phenylhydrazone was carried out using the corresponding standard curve.

### UHPLC analysis of the CoA-thioesters

CoA and CoA-thioesters were detected with Agilent 1290 Infinity II UHPLC using a reversed-phase C18 column (Agilent InfinityLab Poroshell 120 EC-C18 1.9 mm 2.1 mm x 50 mm column). The following acetonitrile gradient in 10 mM potassium phosphate buffer (pH 7) with a flow rate of 0.55 mL min^−1^, was used: from 2 to 8% at 0–2.66 min; from 8 to 30% at 2.66–3.33 min; from 30 to 2% at 3.33–3.68 min; 2% at 3.68–5 min. Retention times were: succinyl-CoA, 0.7 min; CoA, 0.9 min; acetyl-CoA, 1.7 min; propionyl-CoA, 2.4 min. Reaction products and standard compounds were detected by UV absorbance at 260 nm with a 1290 Infinity II diode array detector (Agilent) and the amount of product was calculated from the relative peak area. The identification of the CoA thioesters was based on co-chromatography with standards and analysis of the UV spectra of the products.

### Proteomics analysis

The relative quantification of the cell lysate proteomes of *D. acetivorans* was achieved with data-independent label-free high-definition (HD)MS protein expression analysis on Synapt G2 Si coupled to M-Class UHPLC (Waters Corp., Manchester, UK) following filter-based tryptic digestion using the UniProt *D. acetivorans* databases as previously described (Distler et al., 2016; Wildschütz et al., 2019). In brief, cell lysates were reduced, alkylated and tryptically digested on 10 kDa centrifugal filter devices and prepared for LC–MS at 250 ng μl − 1 in 0.1% formic acid and 5% ACN. For statistical analyses, Progenesis QIP software was used (nonlinear diagnostics/Waters Corp., fixed modification carbamidomethylation, variable modification methionine oxidation, 1 missed cleavage allowed).

## Results and discussion

### Characterization of methylcitrate cycle enzymes in *D. acetivorans*

Although bioinformatic analysis revealed the presence of three *Si*-citrate synthase isozymes in the *D. acetivorans* genome, only a biochemical investigation could reveal their physiological roles. We cloned the citrate synthase gene *AHF97591* into the expression vector pET16b introducing an N-terminal His_10_-tag upon the expression and produced the corresponding protein heterologously in *E. coli*. The characterization of this enzyme confirmed its identification as citrate synthase (Table 1). Moreover, the enzyme was bifunctional and utilized both acetyl-CoA and propionyl-CoA (with oxaloacetate) as substrates (*V_max_* of 26 and 7.4 U mg^−1^ protein, respectively), forming citrate or 2-methylcitrate. Promiscuous citrate/2-methylcitrate synthases are not uncommon and have been characterized in bacterial and eukaryotic organisms (Gerike et al., 1998; Chittori et al., 2011; Maerker et al., 2005). The *D. acetivorans* enzyme shows approximately 3.5-fold higher activity with acetyl-CoA than with propionyl-CoA, but possesses comparable affinities for both CoA-thioesters (*K*_m_ values of 14 and 22 μM, respectively; see Table 1). The enzyme catalyzed also the citrate cleavage reaction, though its activity was low.

**Table 1:** Catalytic properties of the promiscuous citrate/2-methylcitrate synthase AHF97591 from *D. acetivorans*.

Substrate	*V*_max_ (U mg^−1^ protein)	*K*_m_ (μM)	*k*_cat_/*K*_m_ (s^−1^ mM^−1^)
Acetyl-CoA (+oxaloacetate)	26 ± 0.6	14 ± 1.8	1.5
Propionyl-CoA (+oxaloacetate)	7.4 ± 0.3	22 ± 3.5	0.3
Oxaloacetate (+acetyl-CoA)	21 ± 0.7	30 ± 4.4	0.6
Coenzyme A	0.1 ± 0.01	41 ± 11	2
Citrate	0.08 ± 0.003	1,065 ± 241	6.3 ∙ 10^−2^

Specific activities were measured at 55°C; data are mean ± s.d. of three technical replicates.

**Figure 2:**
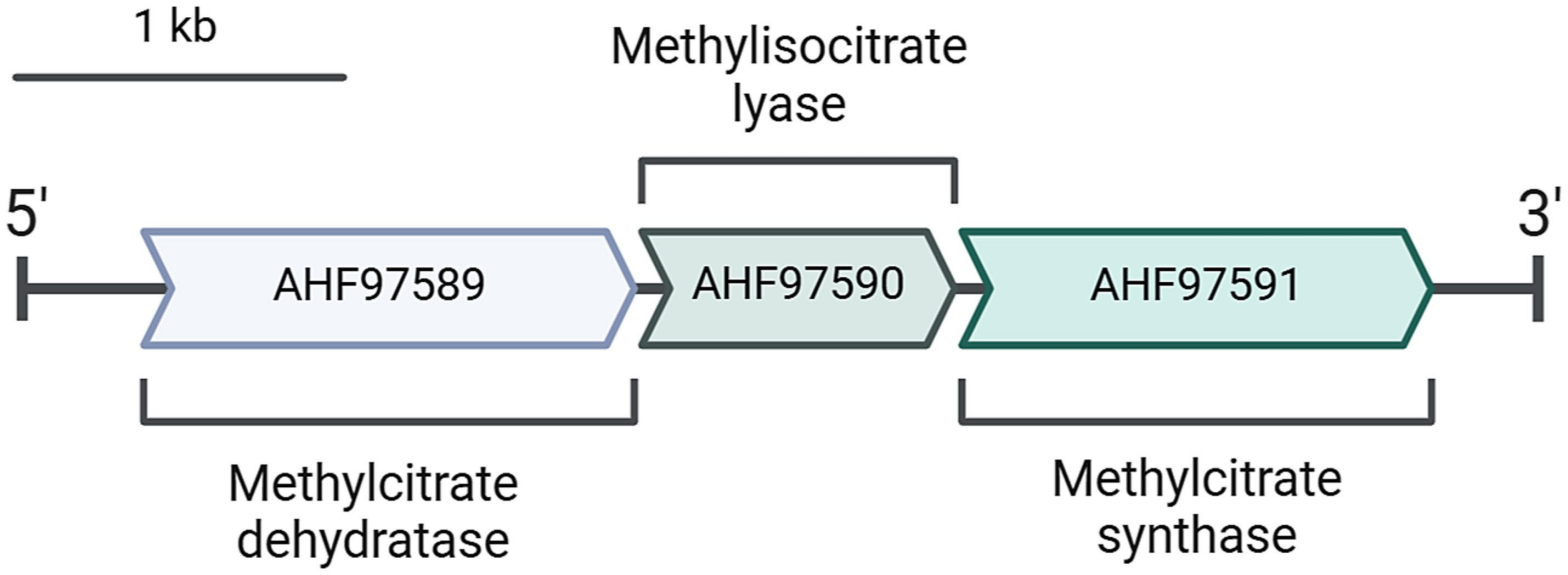
A putative methylcitrate cycle operon present in *D. acetivorans* genome (GenBank: CP007051.1).

The gene encoding the citrate/2-methylcitrate synthase of *D. acetivorans* is located with two other genes (i.e., *AHF97589*, *AHF97590*) in a putative methylcitrate cycle operon that encodes a putative 2-methylcitrate dehydratase and a putative methylisocitrate lyase (Figure 2). In order to check whether these proteins can potentially be involved in the assimilation of propionate, we heterologously expressed the corresponding genes and purified their products. Enzymatic assays performed with the purified AHF97589 provided us qualitative data proving the activity of this protein as a 2-methylcitrate dehydratase (2-methylcitrate ↔ 2-methylaconitate + H_2_O). Indeed, we could measure the formation of 2-methylaconitate and subsequent decrease of 2-methylcitrate via mass spectrometry (MS) analysis (Supplementary Figure 1), thus proving that this enzyme is able to catalyze the second step of the methylcitrate cycle, following the methylcitrate synthase reaction. Furthermore, heterologously produced and purified AHF97590 catalyzed the methylisocitrate lyase reaction (5 U mg^−1^ protein). This enzyme also catalyzed the backwards reaction, e.g., the formation of methylcitrate from pyruvate and succinate, as was shown by the detection of the MS signal at 205 m/z in the negative scan mode (Supplementary Figure 2). Therefore, the enzymes encoded by the putative methylcitrate operon are functional, being capable to catalyze the corresponding reactions of the pathway. Similar clusters were also found in the genomes of other sequenced representatives of *Desulfurella*, *D. amilsii*, and *D. multipotens*.

### Cultivation experiments with propionate as a sole carbon source

**Figure 3:**
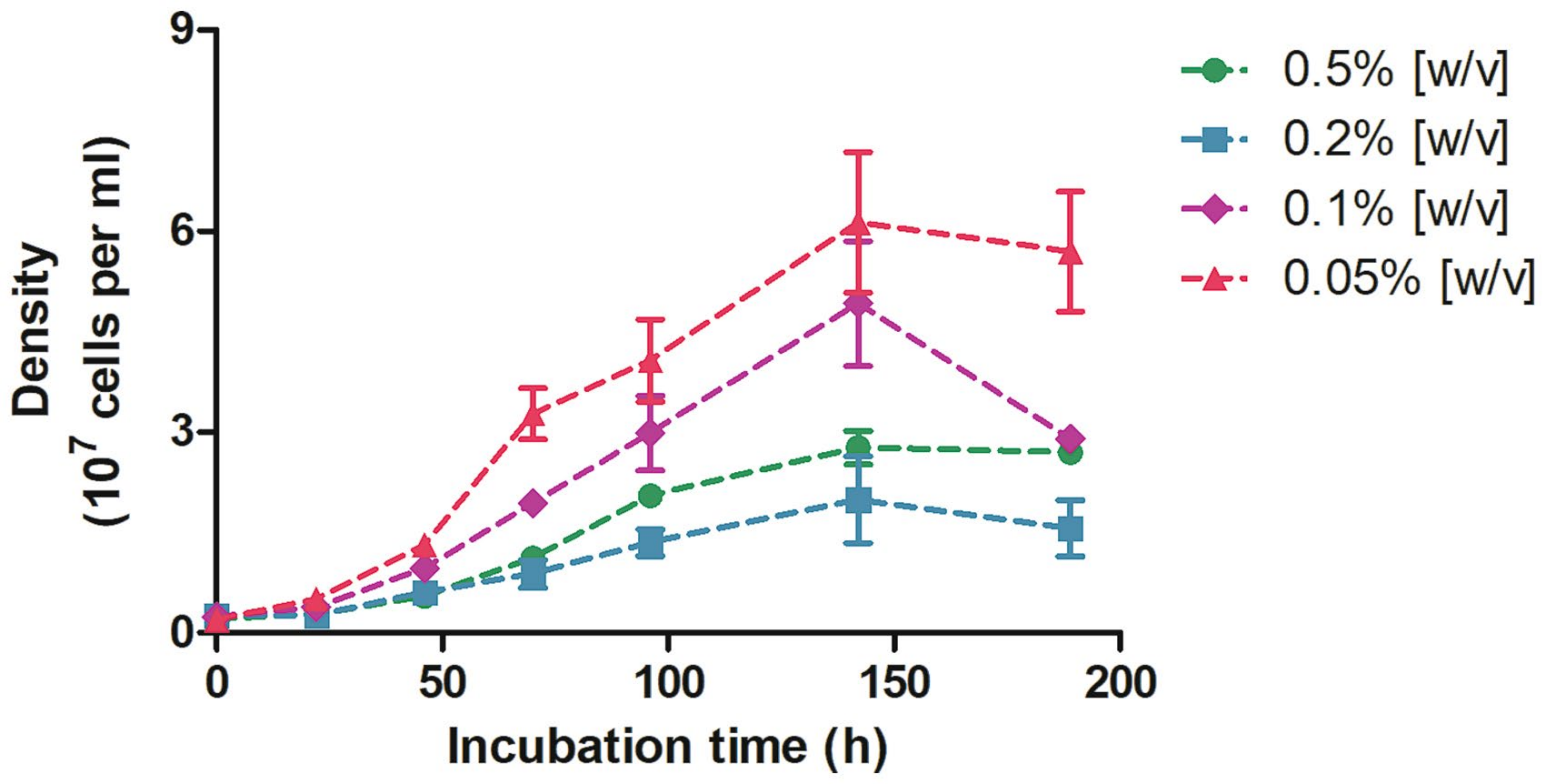
Growth of *D. acetivorans* on propionate (0.5, 0.2, 0.1, and 0.05%, w/v) as a sole carbon source of organic carbon. Data are mean ± s.e.m. of four biological replicates.

In view of the previous observations, we started cultivation experiments to test the ability of *D. acetivorans* to utilize propionate as a sole carbon source. The first signs of reliable growth were observed after 2 weeks of incubation at 55°C in cultures supplemented with 0.5% (w/v) propionate. Specifically, a moderate but significant increment of the cell density (i.e., 10^6^ to 10^7^ cells/ml) could be detected in three different biological replicates, and thereafter reliably after each new inoculation. Subsequently, we grew *D. acetivorans* with different propionate concentrations (0.05, 0.1, 0.2 and 0.5%, w/v) (Figure 3). *D. acetivorans* grew best at 0.05% propionate to a final cell density of 6 · 10^7^ cells/ml with a generation time of 18 h (Figure 3). Growth at higher propionate concentrations may be impaired due to the toxicity of propionate and propionyl-CoA to the cell (Brock and Buckel, 2004).

### Activities of enzyme involved in propionate assimilation

Once we established the capacity of *D. acevitorans* to assimilate propionate, we performed assays to measure the activities of enzymes involved in propionate metabolism. Propionate assimilation starts with its activation to propionyl-CoA. According to our data, propionate activation required propionyl-CoA synthetase activity, whereas activity of (a) CoA-transferase(s) capable to activate propionate using acetyl-CoA or succinyl-CoA as CoA-donors, was more than an order of magnitude down-regulated (Table 2). Accordingly, activity of succinyl-CoA:acetate CoA-transferase capable to activate both acetate and propionate (Pettinato et al., 2022) was also 34-fold down-regulated. Instead of this enzyme, succinyl-CoA synthetase catalyzing succinyl-CoA conversion into succinate in course of the “classical” oTCA cycle (Nelson and Cox, 2017) was up-regulated (Table 2).

**Table 2:** Activities of enzymes involved in propionate metabolism in *D. acetivorans* cells grown under different conditions (in U mg^−1^ protein).

Enzyme/activity	Acetate	Propionate	Autotrophic
TCA/methylcitrate/glyoxylate cycle			
Citrate synthase	44.1 ± 19.4 (*n* = 3)	5.7 ± 6 (*n* = 4)	13.3 ± 8.7 (*n* = 3)
Malate dehydrogenase	30.6 ± 11.7 (*n* = 4)	29.3 ± 14.8 (*n* = 5)	18.4 ± 5.6 (*n* = 3)
Isocitrate dehydrogenase	17.1 ± 2.2 (*n* = 4)	16.4 ± 6.8 (*n* = 3)	8.4 ± 0.24 (*n* = 2)
Isocitrate lyase	<0.02 (*n* = 4)	<0.02 (*n* = 3)	<0.02 (*n* = 1)
Methylcitrate synthase	<0.02 (*n* = 3)	<0.02 (*n* = 3)	<0.02 (*n* = 3)
CoA-transferases			
Succinyl-CoA:acetate	0.24 ± 0.08 (*n* = 7)	0.007 ± 0.003 (*n* = 3)	0.06 ± 0.03 (*n* = 5)
Acetyl-CoA:succinate	0.96 ± 0.11 (*n* = 5)	0.14 ± 0.03 (*n* = 4)	0.6 ± 0.19 (*n* = 3)
Propionyl-CoA:acetate	16 ± 5.5 (*n* = 4)	0.44 ± 0.12 (*n* = 4)	3.6 ± 0.66 (*n* = 3)
Acetyl-CoA:propionate	20.1 ± 2.05 (*n* = 6)	1.1 ± 0.18 (*n* = 4)	5.9 ± 1.15 (*n* = 3)
Propionyl-CoA:succinate	1.05 ± 0.31 (*n* = 5)	0.07 ± 0.03 (*n* = 4)	0.19 ± 0.07 (*n* = 5)
Succinyl-CoA:propionate	0.23 ± 0.07 (*n* = 5)	0.02 ± 0.01 (*n* = 3)	0.07 ± 0.04 (*n* = 5)
Synthetases			
Acetyl-CoA synthetase	0.08 (*n* = 1)	0.06 ± 0.01 (*n* = 3)	0.15 ± 0.04 (*n* = 4)
Propionyl-CoA synthetase	0.01 (*n* = 1)	0.11 ± 0.02 (*n* = 3)	0.27 ± 0.05 (*n* = 3)
Succinyl-CoA synthetase	0.006 ± 0.006 (*n* = 4)	0.08 ± 0.04 (*n* = 4)	0.02 ± 0.02 (*n* = 3)
Acetate kinase	0.06 ± 0.04 (*n* = 3)	0.02 ± 0.01 (*n* = 3)	0.08 ± 0.02 (*n* = 3)
Phosphotransacetylase	0.72 ± 0.16 (*n* = 3)	0.21 ± 0.04 (*n* = 4)	0.31 ± 0.04 (*n* = 4)

Interestingly, the activity of the key enzyme of the methylcitrate cycle, methylcitrate synthase, was not detectable in either propionate-, acetate-, or autotrophically grown cells, suggesting that this cycle is not active in propionate assimilation in *D. acetivorans* (Table 2). An alternative pathway of propionate utilization starts with propionyl-CoA carboxylation catalyzed by propionyl-CoA carboxylase [ATP + propionyl-CoA + HCO_3_^−^ = ADP + phosphate + (*S*)-methylmalonyl-CoA]. Direct measurement of the activity of this enzyme using spectrophotometric or UHPLC assays proved difficult because of the high hydrolysis rates for succinyl-CoA and methylmalonyl-CoA. This hampered the assays based on the formation of methylmalonyl-CoA or succinyl-CoA when the rate of their production was below the rate of hydrolysis. Nonetheless, we were able to obtain qualitative data confirming the activity of propionyl-CoA carboxylase in propionate-grown *D. acetivorans* cell extracts. The assay performed with ^13^C-labeled bicarbonate, propionyl-CoA, and ATP showed the time-dependent formation of [^13^C]methylmalonate, product of the hydrolysis of methylmalonyl-CoA (Supplementary Figure 3). After the start of the reaction, the samples were taken at different time points and analyzed using GC-MS. This experiment proved that propionyl-CoA undergoes a carboxylation step in *D. acetivorans* during propionate assimilation.

### [U-^13^C_3_]propionate isotopologue profiling experiments

**Figure 4:**
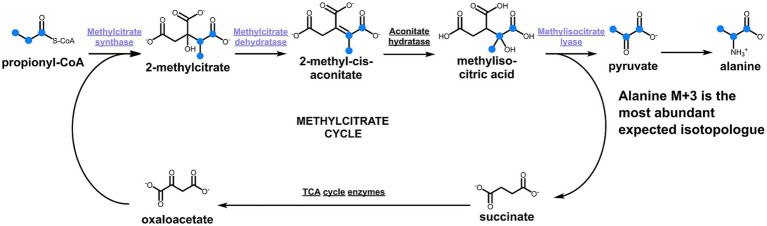
Methylcitrate cycle and the expected labeling patterns of alanine after growth in the presence of [U-^13^C_3_]propionate. The scheme shows ^13^C-labeled metabolites as blue dots.

**Figure 5:**
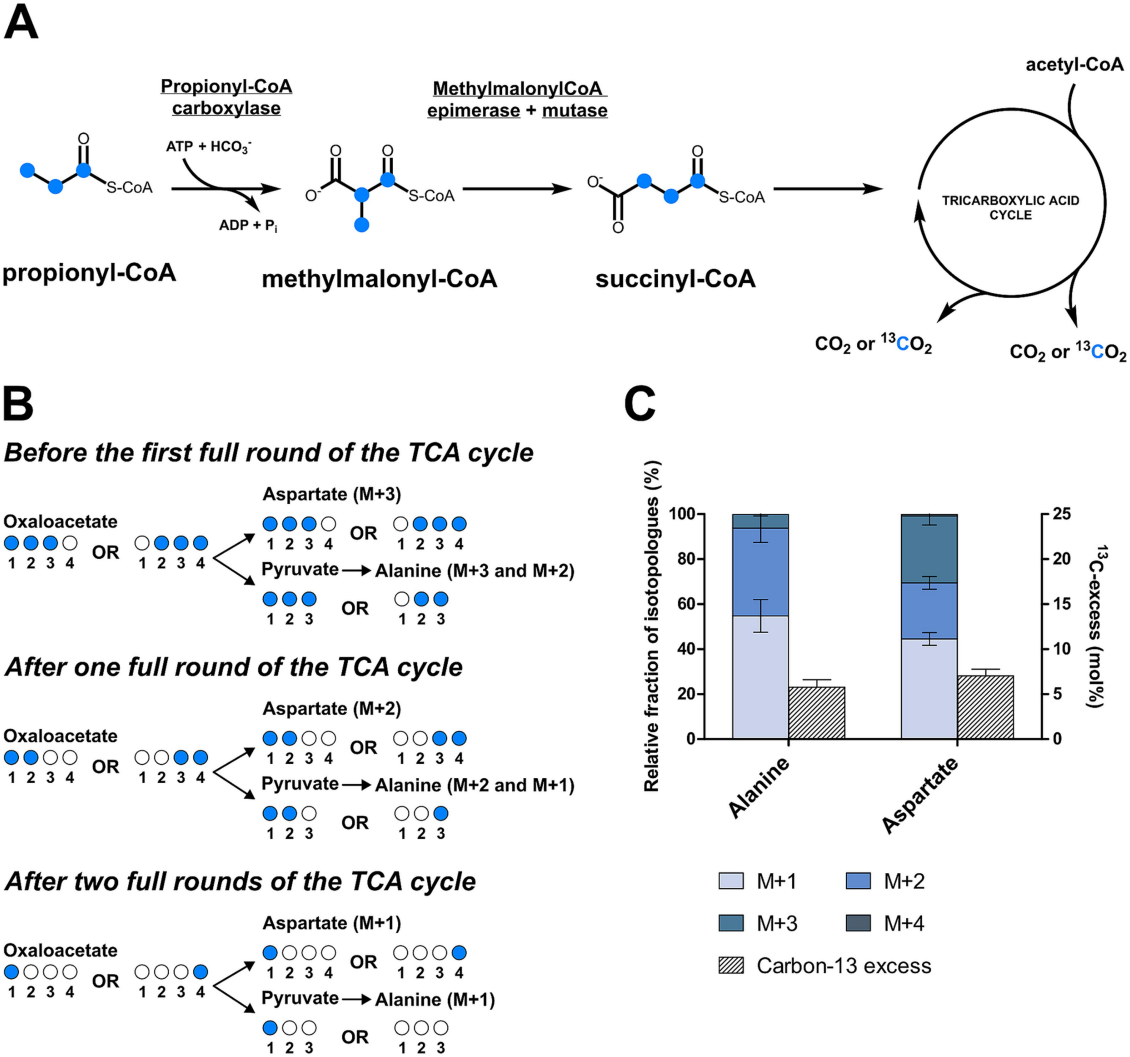
Isotopologues profiling of the amino acids of *D. acetivorans* after cultivation with [U-^13^C_3_]propionate. Schematic representation of the propionate assimilation pathway **(A)** and the expected ^13^C labeling patterns **(B)**. The actual isotopologue profiles of alanine (C_1_-C_3_) and aspartate (C_1_-C_4_) were reported in **(C)**.

To take a closer look at the metabolism of *D. acetivorans*, we performed an isotopologue profiling experiment with [U-^13^C_3_]propionate as a tracer (10% of the total propionate provided). The obtained results were not consistent with the involvement of the methylcitrate cycle in propionate assimilation in *D. acetivorans* (Table 3). Indeed, high proportion of fully labeled alanine (M + 3) was expected after growth with [U-^13^C_3_]propionate (Figure 4) in case of the functioning of the methylcitrate cycle. However, our results did not match these predictions, and fully labeled alanine (M + 3) was detected only in minor amounts, whereas M + 1 and M + 2 alanine isotopologues were the most abundant (55% of M + 1, 45% of M + 2, and 0.5% M + 3 of the total ^13^C-excess of alanine for *D. acetivorans*) (Table 3). Furthermore, the comparison of isotopologues of alanine and aspartate (52 ± 4% of M + 1, 31 ± 3% of M + 2, and 16 ± 2% M + 3 of the total ^13^C-excess of aspartate) suggests that the labeling patterns of alanine were derived from the labeling patterns of aspartate. Indeed, the observed high labeling in C4 of aspartate (Supplementary Table 1) is not expected if oxaloacetate is produced by pyruvate/PEP carboxylation. In contrast, oxaloacetate decarboxylation is consistent with propionate assimilation via the methylmalonyl-CoA pathway, in which propionate is first converted to the C_4_-dicarboxylic acids of the TCA cycle and only then to pyruvate/PEP (Figure 5). However, why is then aspartate not preferentially M + 3 labeled? This can be explained by the high activity of the oTCA cycle during growth on propionate. According to this scheme, propionyl-CoA is carboxylated to (*S*)-methylmalonyl-CoA, undergoes epimerization and isomerization to succinyl-CoA and enters the TCA cycle. From here, succinyl-CoA is converted to oxaloacetate and condenses with acetyl-CoA (produced from decarboxylation of another oxaloacetate molecule to pyruvate and further to acetyl-CoA). The functioning of the oTCA cycle is also accompanied by decarboxylation reactions, causing the progressive loss of ^13^C-label and leading to a pool of oxaloacetate (aspartate) composed of a mixture of different isotopologues (Figure 5). This scheme is supported by the detection in the growth medium of significant amounts of acetate (1.8 ± 0.15 mM) labeled M + 1 and M + 2 in equal ratios.

**Table 3:** The comparison of abundance of enzymes involved in carbon metabolism in *D. acetivorans* proteome during growth with propionate versus acetate.

Enzyme annotation	GenBank accession	Protein expression fold change
Succinyl-CoA synthetase	AHF96946.1/AHF96945.1	8.19 (UP)/10.47 (UP)
Pyruvate synthase	AHF97587.1	5.07 (UP)
Acetate kinase	AHF97582.1	2.54 (DOWN)
Phosphotransacetylase	AHF97583.1	1.54 (DOWN)
Malic enzyme	AHF96745.1	1.16 (DOWN)
Si-Citrate synthase (main)	AHF97477.1	2.93 (DOWN)
Putative Si-citrate synthase (function unknown)	AHF97305.1	1.06 (DOWN)
Malate dehydrogenase	AHF96721.1	3.38 (DOWN)
	AHF97578.1	2.31 (DOWN)
Acetyl-CoA acetyltransferase	AHF96995.1	4.81 (UP)
	AHF96822.1	1.76 (UP)
Putative propionyl-CoA carboxylase		
biotin carboxylase subunit	AHF96739.1	1.94 (UP)
carboxytransferase subunit	AHF96738.1	1.25 (UP)
biotin carboxyl carrier protein	AHF97742.1	1.6 (UP)
Putative acetyl-CoA carboxylase		
biotin carboxylase subunit	AHF96803.1	1.9 (UP)
biotin carboxyl carrier protein	AHF96802.1	1.51 (UP)
Pyruvate carboxylase	AHF96546.1	1.3 (UP)
Succinyl-CoA:acetate CoA-transferase	AHF96498.1	46.4 (DOWN)
Putative propionyl-CoA synthetase (AMP-producing)	AHF96997.1	2.23 (UP)
Putative acyl-CoA synthetase (AMP-producing)	AHF97139.1	1.23 (DOWN)
	AHF97494.1	1.3 (DOWN)
Si-Citrate/2-methylcitrate synthase	AHF97591.1	1.4 (DOWN)
Methylcitrate dehydratase	AHF97589.1	2 (DOWN)
Methylisocitrate lyase	AHF97590.1	1.41 (DOWN)
Methylmalonyl-CoA mutase	AHF96749.1/AHF96748.1	2 (UP)/39.5 (UP)

Data were obtained with LC-HR MS proteomic analysis (for full data set, see Supplementary Excel File, the raw data are available at DOI 10.17879/34908572965), the shortlisted data were visualized in a heatmap shown in the Supplementary Excel File (Table heat). They demonstrate the clear group separation on the protein level.

### Comparative proteomic analysis of *D. acetivorans* cells

Comparison of the proteomes of acetate- and propionate-grown *D. acetivorans* cells further demonstrated that the methylcitrate cycle is not functional during growth on propionate (Table 3). In fact, the three enzymes encoded in the methylcitrate cycle operon (Figure 2) showed no significant regulation between the two growth conditions (Table 3). In contrast, propionyl-CoA carboxylase and methylmalonyl-CoA mutase were up-regulated, consistent with their involvement in propionate utilization.

In the oTCA cycle, succinyl-CoA is further converted to succinate in a succinyl-CoA synthetase reaction. Indeed, succinyl-CoA synthetase subunits (GenBank: AHF96945, AHF96946) were observed with up to 10-fold higher abundance in propionate grown cells. In contrast, succinyl-CoA:acetate CoA-transferase (GenBank: AHF96498) was 46-fold downregulated in propionate-grown cells (Table 3), confirming the results of our enzyme assays (Table 2). This dramatic downregulation of succinyl-CoA:acetate CoA-transferase is in line with the involvement of the oTCA cycle in propionate metabolism: if present, this enzyme would catalyze the transfer of CoA from the synthesized acetyl-CoA to propionate (Pettinato et al., 2022), thus preventing acetyl-CoA oxidation in the oTCA cycle. Therefore, propionate activation relies on AMP-producing propionyl-CoA synthetase, which is consequently 2.2-fold up-regulated in propionate (versus acetate) grown cells (Table 3). In view of these considerations, the upregulation of succinyl-CoA synthetase (succinyl-CoA + P_i_ + ADP ↔ succinate + CoA + ATP) becomes relevant not only for the processing of succinyl-CoA, the product of the carboxylation of propionyl-CoA (via methylmalonyl-CoA), but also for the synthesis of ATP, recovering some of the ATP equivalents spent for the activation of propionate.

Pyruvate synthase (GenBank: AHF97587) required for pyruvate conversion to acetyl-CoA was 5-fold up-regulated, while *Si*-citrate synthase (GenBank: AHF97477) was 3-fold downregulated (but still highly active) during propionate growth (Table 3).

When we started this study, we were guided by the bioinformatics data and expected to show the involvement of the methylcitrate cycle in propionate utilization in *D. acetivorans*. This would have been the first example of this pathway being used by a strictly anaerobic bacterium. Instead, we found a pathway that starts with propionyl-CoA carboxylation in a propionyl-CoA carboxylase reaction, followed by conversion of the product to the C_4_-dicarboxylates of the TCA cycle and their decarboxylation to pyruvate/PEP and then to acetyl-CoA, which is further degraded in the oTCA cycle. This highlights the key role of the TCA cycle in *D. acetivorans* metabolism. The main adaptation to the use of propionate was in its activation pathway, while the other enzymes were not significantly regulated. It seems that the main strategy of *D. acetivorans* is to avoid the regulation of the central metabolism as long as possible and to make only the absolutely necessary adaptations (i.e., activation mechanism). The direction of metabolism is mainly determined by the concentration of substrates available in the medium (Steffens et al., 2021). The functions of the methylcitrate cycle cluster in *Desulfurella* spp. are not clear. It could have been recently obtained by a *Desulfurella* ancestor and not yet gained a function, or it could be active under certain growth conditions, or could be left over from ancestors that used the cycle for propionate oxidation. In our work, we could not observe any evidence of the activity of this pathway, showing that bioinformatic predictions should be taken with caution and require experimental confirmation. Even the detection of activities in purified enzymes does not mean that these enzymes are active *in vivo* and their role in metabolism should be investigated biochemically.

## Data Availability

The datasets presented in this study can be found in online repositories. The names of the repository/repositories and accession number(s) can be found in the article/Supplementary material.
