# Supplementary material for: Propionate metabolism in Desulfurella acetivorans
Source: Front Microbiol. 2025 Feb 12;16:1545849. doi: 10.3389/fmicb.2025.1545849 (PMC11861179; doi:10.3389/fmicb.2025.1545849)
Supplement: Supplementary file 2 [file Data_Sheet_1.pdf]

Supplementary Material

Supplementary Figures

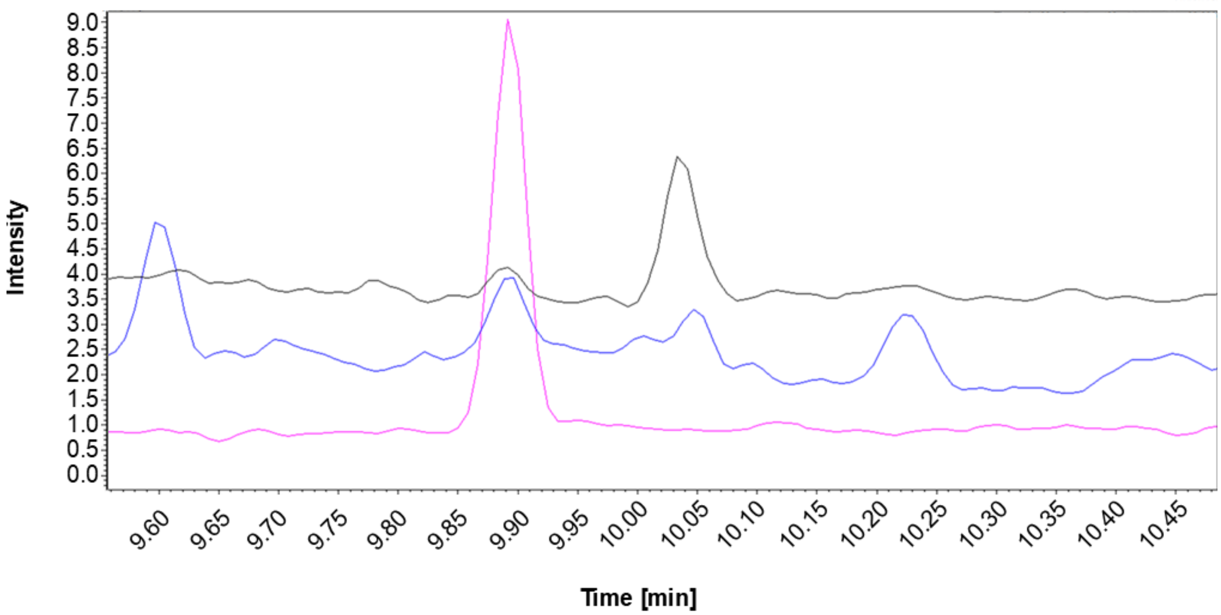

Supplementary Figure 1. GC-MS detection of methylaconitate formed from methylcitrate in a reaction catalyzed by methylcitrate dehydratase AHF97589. The chromatogram shows the formation of methylaconitate in course of two different assays (black and blue) after 10 minutes from the start of the reaction, with retention times compared to methylaconitate reference substance (pink). Please note that the reference substance used was methyl-*trans*-aconitate, but which stereoisomer is actually formed as a result of the activity of the 2-methylcitrate dehydratase is not clear. Indeed, isomerization could occur in the course of the derivatization and injection steps of the GC-MS analysis.

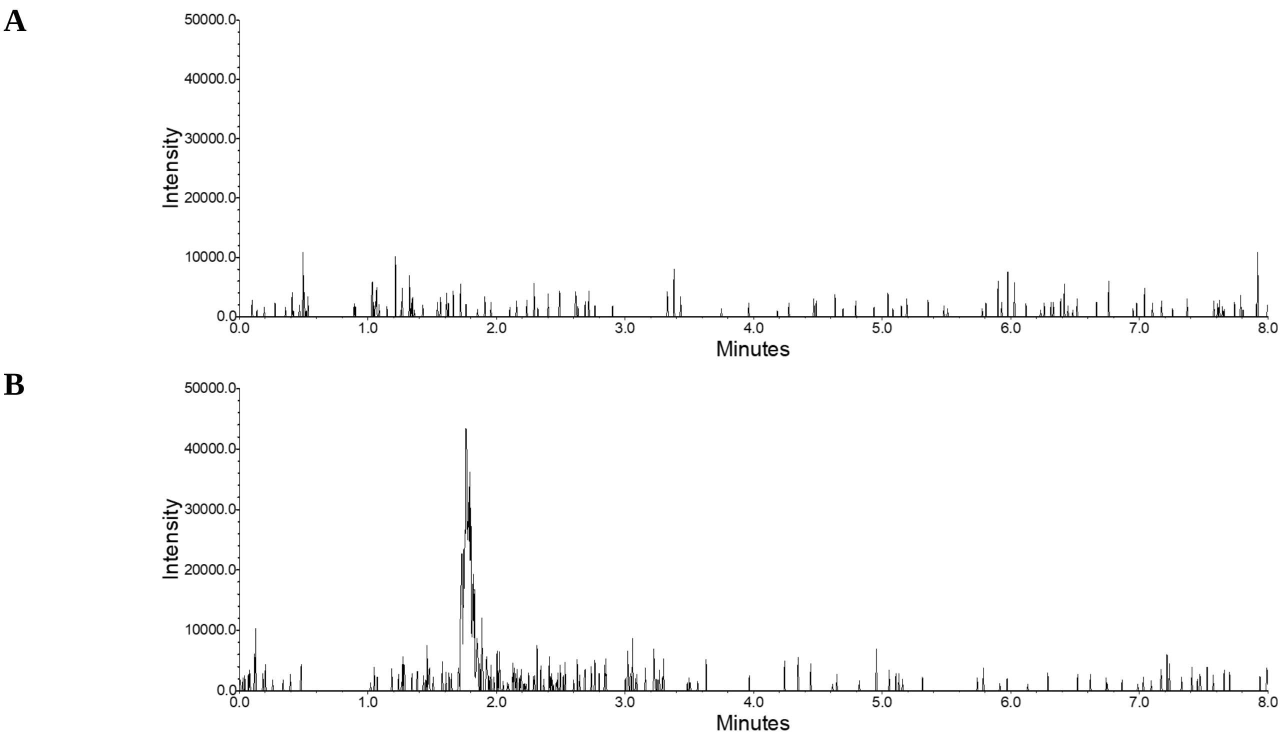

Supplementary Figure 2. LC-MS detection of methylisocitrate formed from succinate and pyruvate in a reaction catalyzed by methylisocitrate lyase AHF97590 after 0 (A) and 60 min of incubation (B). The intensity of the MS signal at 205 m/z in the negative scan is shown.

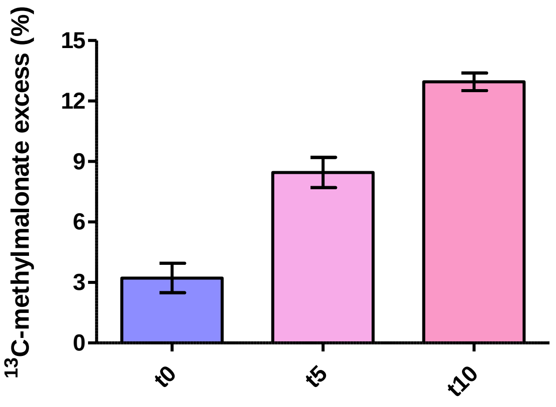

Supplementary Figure 3. [1-^{13}C]Methylmalonate produced in the propionyl-CoA carboxylase reaction in the presence of $NaH^{13}CO_3$ catalyzed by cell extracts of propionate-grown *D. acetivorans* cells. The blue column represents the sample taken immediately after the start of the reaction (t0), pink column after 5 minutes (t5), purple column after 10 minutes (t10).

Supplementary Tables.

Supplementary Table 1. ^{13}C-Enrichments in amino acids from *D. acetivorans* grown on [U-$^{13}C_3$]propionate, labelling 10% of the total 0.05 % (w/v) and N_2:CO_2 (80:20, v/v) as the gas phase.

Amino acid or organic acid	Isotopologues	Replicate 1		Replicate 2		Replicate 3		Replicate 4		Combined	
		Average	St.dev	Average	St.dev	Average	St.dev	Average	St.dev	Average	St.dev
Ala(C2-3)	M+1	4.28 %	0.12 %	6.19 %	0.10 %	5.99 %	0.04 %	5.72 %	0.10 %	5.55 %	0.87%
	M+2	3.50 %	0.03 %	4.89 %	0.03 %	5.12 %	0.01 %	4.78 %	0.05 %	4.57 %	0.73%
Ala(C1-3)	M+1	5.15 %	0.09 %	7.04 %	0.15 %	6.68 %	0.13 %	6.26 %	0.14 %	6.28 %	0.83%
	M+2	3.41 %	0.02 %	4.82 %	0.06 %	5.02 %	0.05 %	4.66 %	0.02 %	4.48 %	0.73%
	M+3	0.58 %	0.00 %	0.75 %	0.00 %	0.72 %	0.02 %	0.75 %	0.01 %	0.70 %	0.08%
Asp(C2-4)	M+1	6.96 %	0.17 %	8.12 %	0.10 %	7.43 %	0.33 %	7.27 %	0.24 %	7.44 %	0.53%
	M+2	3.71 %	0.05 %	4.70 %	0.02 %	4.65 %	0.12 %	4.53 %	0.13 %	4.40 %	0.48%
	M+3	1.92 %	0.03 %	2.51 %	0.02 %	2.62 %	0.03 %	2.53 %	0.02 %	2.39 %	0.32%
Asp(1-4)	M+1	6.27 %	0.12 %	7.23 %	0.22 %	6.68 %	0.22 %	6.63 %	0.08 %	6.70 %	0.42%
	M+2	3.13 %	0.06 %	4.02 %	0.07 %	3.94 %	0.06 %	3.95 %	0.07 %	3.76 %	0.43%
	M+3	3.59 %	0.08 %	4.73 %	0.02 %	4.91 %	0.04 %	4.69 %	0.02 %	4.48 %	0.60%
	M+4	0.06 %	0.02 %	0.11 %	0.03 %	0.13 %	0.03 %	0.12 %	0.02 %	0.11 %	0.04%
Glu(C2-5)	M+1	7.10 %	0.07 %	8.96 %	0.09 %	8.37 %	0.29 %	8.38 %	0.14 %	8.20 %	0.80%
	M+2	4.50 %	0.11 %	6.08 %	0.03 %	5.92 %	0.07 %	6.03 %	0.09 %	5.63 %	0.76%
	M+3	2.98 %	0.03 %	3.54 %	0.07 %	3.69 %	0.08 %	3.27 %	0.05 %	3.37 %	0.32%
	M+4	0.00 %	0.00 %	0.00 %	0.00 %	0.00 %	0.00 %	0.00 %	0.00 %	0.00 %	0.00%
Glu(C1-5)	M+1	6.42 %	0.03 %	8.50 %	0.31 %	7.94 %	0.06 %	7.83 %	0.41 %	7.67 %	0.91%
	M+2	4.44 %	0.06 %	5.93 %	0.07 %	5.88 %	0.05 %	5.83 %	0.22 %	5.52 %	0.73%
	M+3	3.23 %	0.06 %	3.84 %	0.04 %	4.09 %	0.13 %	3.93 %	0.04 %	3.77 %	0.38%
	M+4	0.16 %	0.01 %	0.25 %	0.03 %	0.23 %	0.03 %	0.22 %	0.03 %	0.22 %	0.05%
	M+5	0.02 %	0.01 %	0.03 %	0.00 %	0.03 %	0.01 %	0.03 %	0.01 %	0.03 %	0.01%
Gly	M+1	4.30 %	0.12 %	6.33 %	0.08 %	6.23 %	0.05 %	5.96 %	0.08 %	5.71 %	0.95%

	M+2	2.06 %	0.07 %	3.37 %	0.04 %	3.36 %	0.01 %	3.29 %	0.01 %	3.02 %	0.64%
Ile	M+1	4.13 %	0.25 %	5.60 %	0.19 %	5.30 %	0.15 %	4.78 %	0.15 %	4.95 %	0.67%
	M+2	3.90 %	0.04 %	5.00 %	0.11 %	5.21 %	0.03 %	4.82 %	0.07 %	4.73 %	0.58%
	M+3	7.53 %	0.05 %	7.64 %	0.03 %	7.64 %	0.06 %	7.39 %	0.06 %	7.55 %	0.13%
	M+4	0.34 %	0.02 %	0.51 %	0.04 %	0.44 %	0.02 %	0.40 %	0.01 %	0.42 %	0.07%
	M+5	0.27 %	0.01 %	0.36 %	0.01 %	0.40 %	0.00 %	0.35 %	0.01 %	0.34 %	0.05%
Leu	M+1	10.52 %	0.07 %	15.71 %	0.24 %	15.24 %	0.17 %	13.94 %	0.18 %	13.85 %	2.35%
	M+2	5.92 %	0.08 %	8.84 %	0.06 %	9.20 %	0.07 %	8.33 %	0.04 %	8.07 %	1.48%
	M+3	0.79 %	0.03 %	1.31 %	0.01 %	1.29 %	0.04 %	1.15 %	0.06 %	1.13 %	0.25%
	M+4	0.58 %	0.03 %	0.52 %	0.00 %	0.59 %	0.02 %	0.56 %	0.02 %	0.56 %	0.04%
	M+5	0.05 %	0.01 %	0.04 %	0.00 %	0.06 %	0.00 %	0.05 %	0.01 %	0.05 %	0.01%
Lys	M+1	8.97 %	0.49 %	11.38 %	0.31 %	10.87 %	0.16 %	10.66 %	0.15 %	10.47 %	1.08%
	M+2	6.13 %	0.21 %	8.40 %	0.02 %	8.26 %	0.23 %	8.08 %	0.18 %	7.72 %	1.08%
	M+3	3.40 %	0.05 %	4.33 %	0.10 %	4.51 %	0.04 %	4.28 %	0.03 %	4.13 %	0.50%
	M+4	0.03 %	0.03 %	0.26 %	0.06 %	0.24 %	0.02 %	0.20 %	0.04 %	0.18 %	0.11%
	M+5	0.00 %	0.00 %	0.02 %	0.01 %	0.04 %	0.01 %	0.01 %	0.02 %	0.02 %	0.02%
	M+6	0.00 %	0.00 %	0.00 %	0.00 %	0.00 %	0.00 %	0.00 %	0.00 %	0.00 %	0.00%
Phe	M+1	14.28 %	0.40 %	19.23 %	0.53 %	18.12 %	0.40 %	17.06 %	0.09 %	17.17 %	2.15%
	M+2	8.60 %	0.16 %	12.32 %	0.13 %	12.52 %	0.18 %	11.94 %	0.18 %	11.35 %	1.85%
	M+3	2.54 %	0.02 %	4.02 %	0.09 %	3.83 %	0.09 %	3.65 %	0.04 %	3.51 %	0.66%
	M+4	0.73 %	0.02 %	1.13 %	0.02 %	1.16 %	0.02 %	1.07 %	0.02 %	1.02 %	0.20%
	M+5	0.22 %	0.03 %	0.36 %	0.04 %	0.36 %	0.02 %	0.32 %	0.01 %	0.32 %	0.07%
	M+6	0.01 %	0.01 %	0.01 %	0.01 %	0.03 %	0.01 %	0.03 %	0.02 %	0.02 %	0.02%
	M+7	0.00 %	0.00 %	0.00 %	0.00 %	0.00 %	0.00 %	0.00 %	0.00 %	0.00 %	0.00%
	M+8	0.00 %	0.00 %	0.00 %	0.00 %	0.00 %	0.00 %	0.00 %	0.00 %	0.00 %	0.00%

	M+9	0.00 %	0.00 %	0.00 %	0.00 %	0.00 %	0.00 %	0.00 %	0.00 %	0.00 %	0.00%
Pro	M+1	6.08 %	0.15 %	8.11 %	0.17 %	7.27 %	0.15 %	7.13 %	0.17 %	7.15 %	0.85%
	M+2	3.48 %	0.05 %	5.12 %	0.15 %	5.07 %	0.10 %	5.05 %	0.06 %	4.68 %	0.81%
	M+3	3.27 %	0.06 %	3.92 %	0.04 %	4.15 %	0.07 %	3.90 %	0.03 %	3.81 %	0.38%
	M+4	0.31 %	0.01 %	0.37 %	0.01 %	0.37 %	0.01 %	0.34 %	0.04 %	0.35 %	0.03%
	M+5	0.00 %	0.00 %	0.00 %	0.00 %	0.01 %	0.00 %	0.01 %	0.01 %	0.00 %	0.01%
Ser	M+1	6.77 %	0.12 %	9.12 %	0.25 %	8.93 %	0.28 %	8.86 %	0.19 %	8.42 %	1.12%
	M+2	2.89 %	0.14 %	4.29 %	0.10 %	4.43 %	0.11 %	4.27 %	0.06 %	3.97 %	0.73%
	M+3	1.22 %	0.06 %	1.76 %	0.04 %	1.80 %	0.06 %	1.77 %	0.01 %	1.64 %	0.28%
Thr	M+1	7.03 %	0.25 %	8.14 %	0.18 %	6.77 %	0.30 %	6.69 %	0.73 %	7.16 %	0.76%
	M+2	3.59 %	0.12 %	4.60 %	0.14 %	4.70 %	0.11 %	4.61 %	0.26 %	4.37 %	0.55%
	M+3	3.02 %	0.05 %	4.21 %	0.02 %	4.37 %	0.07 %	4.13 %	0.08 %	3.93 %	0.62%
	M+4	0.00 %	0.00 %	0.00 %	0.00 %	0.00 %	0.00 %	0.00 %	0.00 %	0.00 %	0.00%
Tyr	M+1	14.34 %	0.03 %	19.02 %	0.27 %	18.22 %	0.31 %	17.79 %	0.23 %	17.34 %	2.08%
	M+2	9.28 %	0.19 %	12.75 %	0.05 %	12.86 %	0.43 %	12.45 %	0.17 %	11.83 %	1.72%
	M+3	2.65 %	0.06 %	3.87 %	0.08 %	3.99 %	0.13 %	3.87 %	0.09 %	3.59 %	0.64%
	M+4	0.63 %	0.02 %	1.05 %	0.04 %	1.10 %	0.05 %	1.06 %	0.04 %	0.96 %	0.23%
	M+5	0.22 %	0.01 %	0.39 %	0.03 %	0.37 %	0.02 %	0.36 %	0.03 %	0.34 %	0.08%
	M+6	0.03 %	0.01 %	0.03 %	0.02 %	0.03 %	0.01 %	0.05 %	0.00 %	0.04 %	0.01%
	M+7	0.00 %	0.00 %	0.00 %	0.00 %	0.00 %	0.00 %	0.00 %	0.00 %	0.00 %	0.00%
	M+8	0.00 %	0.00 %	0.00 %	0.00 %	0.00 %	0.00 %	0.00 %	0.00 %	0.00 %	0.00%
	M+9	0.00 %	0.00 %	0.03 %	0.04 %	0.01 %	0.01 %	0.01 %	0.01 %	0.01 %	0.02%
Val	M+1	8.35 %	0.10 %	11.70 %	0.39 %	10.92 %	0.20 %	10.37 %	0.17 %	10.33 %	1.45%
	M+2	5.89 %	0.10 %	8.65 %	0.06 %	9.04 %	0.05 %	8.27 %	0.12 %	7.96 %	1.42%
	M+3	0.60 %	0.06 %	0.96 %	0.03 %	0.90 %	0.03 %	0.84 %	0.00 %	0.83 %	0.16%
	M+4	0.70 %	0.02 %	0.55 %	0.01 %	0.64 %	0.01 %	0.60 %	0.01 %	0.62 %	0.06%

	M+5	0.01 %	0.00 %	0.02 %	0.01 %	0.01 %	0.00 %	0.02 %	0.00 %	0.01 %	0.01%
Acetate	M+1	1.01 %	0.09 %	3.50 %	0.05 %	3.73 %	0.22 %	3.21 %	0.04 %	2.86 %	1.26%
	M+2	1.26 %	0.04 %	2.74 %	0.04 %	3.21 %	0.26 %	2.87 %	0.07 %	2.52 %	0.87%
Propionate	M+1	0.18 %	0.16 %	0.41 %	0.10 %	0.10 %	0.09 %	0.16 %	0.25 %	0.21 %	0.21%
	M+2	0.37 %	0.15 %	0.64 %	0.04 %	0.47 %	0.06 %	0.46 %	0.17 %	0.48 %	0.15%
	M+3	8.27 %	0.62 %	8.50 %	0.23 %	7.84 %	0.23 %	8.00 %	0.66 %	8.15 %	0.52%

Supplementary Table 2. The gradient used for the separation of tricarboxylic acids using Waters Premier UPLC system. Buffer A, 50 mM ammonium formate and 0.9% formic acid in water (pH 2.9), buffer B, 0.9% formic acid in acetonitrile, and buffer C, 0.9% formic acid in water.

Time, min	Buffer A, %	Buffer B, %	Buffer C, %
**0.0**	0	0	100
**1.4**	0	0	100
**1.5**	60	0	40
**5.0**	60	40	0
**7.0**	60	40	0
**7.1**	0	0	100
**8.1**	0	0	100
